# Immunopathogenic Mechanisms in Connective Tissue Disease-Associated Interstitial Lung Disease: Incessant Loop of Immunity to Fibrosis

**DOI:** 10.3390/ijms262412126

**Published:** 2025-12-17

**Authors:** Jae Ha Lee, Ji Hoon Jang, Sunggun Lee, Minyoung Her

**Affiliations:** 1 Division of Pulmonology and Critical Care Medicine, Inje University Haeundae Paik Hospital, Inje University College of Medicine, Busan 48108, Republic of Korea; anilleus@naver.com (J.H.L.); saturn80396@gmail.com (J.H.J.); 2 Division of Rheumatology, Department of Internal Medicine, Inje University Haeundae Paik Hospital, Inje University College of Medicine, Busan 48108, Republic of Korea; sglee.ac@gmail.com

**Keywords:** autoimmunity, interstitial lung disease, pulmonary fibrosis, connective tissue diseases, rheumatoid arthritis, systemic sclerosis

## Abstract

Connective tissue disease-associated interstitial lung disease (CTD-ILD) represents a significant cause of morbidity and mortality. It is characterized by the progressive convergence of chronic inflammation, immune dysregulation, and fibrotic remodeling in the lung parenchyma. While often conceptualized through a model of idiopathic pulmonary fibrosis (IPF), CTD-ILD is fundamentally an immune-driven pathology with distinct inflammatory mechanisms in which adaptive immunity plays a profound role. This narrative review explores the “inflammation–immunity–fibrosis continuum” in CTD-ILD, elaborating the intricate cellular and molecular pathways that distinguish it from IPF. We highlight the central role of persistent T-cell responses and B-cell dysregulation, which often occur within organized tertiary lymphoid structures in the lung. This review examines how these immune processes are propagated by multiple cytokine pathways, including the TGF-β/SMAD, JAK/STAT, and phosphodiesterase-4 signaling pathways, which serve as crucial links between inflammation and fibrosis. This distinct immune mechanism in CTD-ILD explains why immunomodulatory agents are a cornerstone of CTD-ILD treatment, in contrast to their limited efficacy in IPF, and emphasizes the current paradigm of combining immunosuppression with antifibrotic drugs to target the dual drivers of the disease.

## 1. Introduction

The pulmonary manifestations of connective tissue diseases (CTDs) represent one of the most complex and challenging interfaces between rheumatology and pulmonology. Interstitial lung diseases (ILDs) associated with CTD arise from the convergence of chronic inflammation, immune dysregulation, and progressive fibrosis in the lung. Lung involvement in connective tissue disease-associated interstitial lung disease (CTD-ILD) results from recurrent alveolar epithelial injury with aberrant immune responses and impaired healing [1]. An initial insult to the delicate alveolar epithelium, which include immune-mediated damage, viral injury, or environmental exposure, triggers an innate immune reaction and the recruitment of inflammatory cells into the alveolar space [2,3,4]. Instead of normal resolution, a cycle of chronic inflammation and autoimmunity ensues, leading to dysfunctional repair in which fibroblasts proliferate and deposit excess collagen in the interstitium [5]. Over time, persistent immune activation and fibrogenesis form a continuum, each reinforcing the other; inflammation fails to fully resolve and instead promotes fibrotic remodeling, while the expanding scar tissue can release damage signals that perpetuate immune activation [6,7]. This inflammation–immunity–fibrosis continuum underlies progressive lung function decline under these conditions [8]. Genetic predispositions and autoantibodies in CTD further skew the balance toward autoimmunity and fibrogenesis in CTD-ILD [9,10,11].

While various rheumatic diseases may involve different primary pathogenic pathways, for instance, systemic sclerosis is characterized predominantly by fibrosis, whereas synovitis in rheumatoid arthritis (RA) is driven mainly by innate and adaptive immunity; these diseases often exhibit convergent histological patterns, such as persistent alveolar inflammation, in the context of ILD [12,13]. These findings suggest that despite diverse initial triggers, common downstream effector mechanisms contribute to lung pathology.

This review synthesizes current evidence on the immunopathogenic mechanisms underlying CTD-ILD, spanning the continuum from alveolar epithelial injury to innate and adaptive immune activation and fibroblast-driven matrix remodeling. We highlight recent advances in understanding how immune dysregulation drives fibrosis in CTD-ILD, offering a mechanistic foundation for future therapies. By integrating perspectives from rheumatology, pulmonology, and immunology, we aim to provide a comprehensive picture of how immune-driven lung injury in CTD leads to progressive fibrosis and how these mechanisms both overlap with and diverge from those in idiopathic pulmonary fibrosis (IPF).

## 2. Immunopathogenesis of CTD-Associated ILD

**Figure 1:**
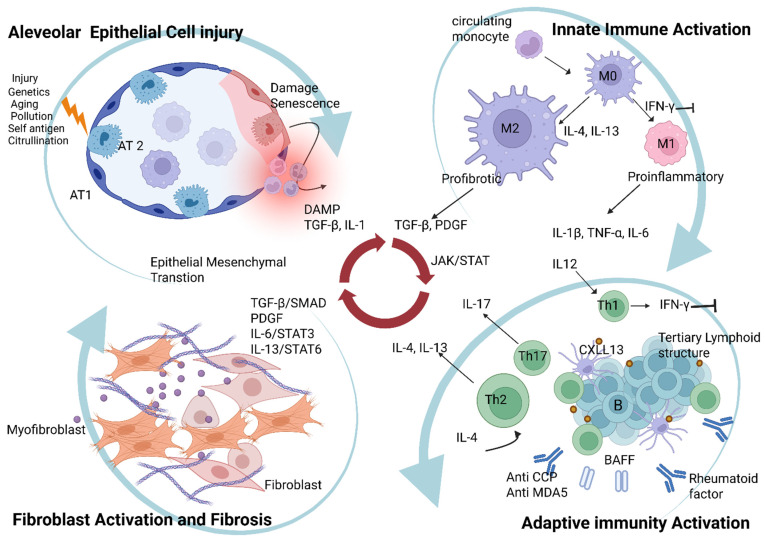
Schematic of the immunopathogenesis involved in CTD-ILD: Inflammation–Immunity–Fibrosis Continuum. This is a self-reinforcing and intertwined cycle. Initial epithelial injury triggers the immune system, which in turn activates fibroblasts and causes fibrosis. This fibrosis further damages epithelial cells, perpetuating the cycle and driving progressive disease. This process consists of the following key steps with multidirectional interactions: (1) Alveolar epithelial cell injury and dysfunction: Initial injury to alveolar epithelial cells by genetics, environmental factors, immune complexes and cytotoxic T cells. This damage releases DAMPs, leading to inflammation, and can cause some epithelial cells to transform into fibroblasts via epithelial–mesenchymal transition. (2) Innate immune activation: Alveolar macrophages are activated by damage signals. They differentiate into proinflammatory M1 or profibrotic M2 macrophages (skewed to M2), releasing cytokines that promote either increased inflammation or fibroblast activation. (3) Adaptive immunity and autoimmunity: The immune response is sustained by T cells (Th2 and Th17 cells) and B cells. B cells form TLSs in the lung, continuously producing autoantibodies that fuel the disease. (4) Fibroblast activation leading to irreversible fibrosis: Immune and epithelial signals, particularly TGF-β and PDGF, activate fibroblasts. These cells differentiate into myofibroblasts, which produce excessive extracellular matrix, leading to scarring and irreversible lung damage. Created in BioRender. Her, m. (2025) https://BioRender.com/mt7mtr1 (accessed on 15 December 2025).

The immunopathogenesis of CTD-ILD can be understood as a multistep process involving (1) alveolar epithelial cell injury and dysfunction, (2) innate immune activation (especially involving alveolar macrophages and related cytokines), (3) adaptive immunity and autoimmunity (T and B cells), and (4) fibroblast activation leading to irreversible fibrosis. These components are tightly interwoven in a multidirectional feedback loop—essentially an ‘inflammation–immunity–fibrosis continuum’ (Figure 1). Here, we detail each aspect and the key molecular mediators involved.

### 2.1. Alveolar Epithelial Cell Injury and Dysfunctional Repair

The development of pulmonary fibrosis is typically initiated by recurrent damage to the alveolar epithelium [14,15]. The alveolar surface, which is lined by type I pneumocytes (AT1) for gas exchange and type II pneumocytes (AT2) for surfactant production and regeneration, is a primary target of immune-mediated damage in both CTD and IPF [16]. In genetically susceptible individuals, even minor insults can trigger the excessive apoptosis or senescence of AT2 cells, impairing their ability to regenerate the epithelium [17,18]. This results in a denuded or leaky alveolar-capillary barrier, which releases danger signals and proinflammatory mediators into the lung microenvironment [19].

#### 2.1.1. Mechanisms of Epithelial Injury in CTD-ILD

Alveolar epithelial cell injury in CTD-ILD can be caused by not only known traditional factors such as infection, air pollution, and aging but also various cytotoxic immune processes. These include attacks by CD8+ T cells or natural killer cells on self-antigens or the deposition of autoantibodies and complement in the alveolar wall [1,16,20].

In RA-ILD, environmental triggers such as infection and pollution, conditions such as periodontitis and changes in the intestinal microbiome can lead to disrupted mucosal immunity and subsequent mucosal injury [21,22,23]. This injury can lead to the formation of immune complexes involving anti-citrullinated protein (anti-CCP) antibodies or rheumatoid factor. These complexes subsequently localize to the lung, where they activate complement and recruit inflammatory cells such as neutrophils and macrophages, ultimately damaging the alveoli [21,24]. The presence of citrullinated peptides and peptidyl arginine deiminase (PAD) enzymes—factors also involved in RA synovitis—is a distinct feature of RA-ILD [25,26,27]. These molecules may directly injure alveolar macrophages, contributing to the usual interstitial pneumonia (UIP) pattern commonly seen in RA-ILD, in contrast to the nonspecific interstitial pneumonia (NSIP) pattern typical of other CTDs. Local citrullination, often driven by factors such as smoking, likely contributes to the unique fibrotic lung microenvironment in RA-ILD [25,26]. The lung may play a role as the initial immunological site that incites RA synovitis in patients who first present with lung manifestations [11,28,29].

In systemic sclerosis, early vascular injury precedes and accompanies epithelial damage. Anti-endothelial cell antibodies and immune complexes targeting the microvasculature lead to endothelial dysfunction and increased vascular permeability, subsequently injuring the epithelium [30]. The resulting pattern, characterized by ground-glass opacities in the lung bases, reflects this unique vascular-epithelial injury and differs markedly from the subpleural honeycomb pattern typical of IPF [16].

#### 2.1.2. Dysfunctional Epithelial Repair and Fibrosis

Dysfunctional healing of the epithelium is a hallmark of the initiation of fibrosis [31]. Instead of undergoing orderly re-epithelialization, injured or senescent AT2 cells fail to properly repopulate the denuded basement membrane; they may also adopt abnormal, profibrotic phenotypes. These injured cells release damage-associated molecular patterns (DAMPs) and fibrogenic cytokines such as transforming growth factor-β (TGF-β) and IL-1 [31,32]. DAMPs, which can be recognized by pattern recognition receptors on neighboring cells, trigger a local inflammatory response that perpetuates a cycle of inflammation and fibrosis [7,31]. These DAMPs can activate macrophages and fibroblasts through a TGF-β mediated signaling loop, illustrating how injured epithelial cells initiate and amplify an immune-fibrotic circuit [33,34,35]. Citrullinated proteins—generated through inflammatory post-translational modification of self-antigens—are a particularly relevant class of DAMPs in RA-ILD [36]. They trigger innate immune activation through Toll-like receptors (TLRs) and enhance autoimmunity by driving anti-CCP antibody responses, which may contribute to the high prevalence of the UIP-pattern in RA-ILD [36,37,38].

There is also evidence that alveolar epithelial-mesenchymal transition can occur [39,40]. Under the influence of TGF-β, some epithelial cells downregulate epithelial marker expression and upregulate mesenchymal gene (e.g., SNAIL, N-cadherin, alpha-smooth muscle actin, and vimentin) expression, directly contributing to the fibroblast pool [41,42,43]. The presence of these hybrid-phenotype cells in lung tissue suggests that dysfunctional epithelial cells can directly transform into collagen-secreting fibroblasts, providing a direct cellular link between epithelial injury and fibrotic scarring [44,45]. This highlights that the epithelium is not a passive bystander but a central driver of the disease.

#### 2.1.3. Genetic Predisposition and Senescence

Telomere shortening and epithelial senescence are central contributors to defective repair in fibrotic lung disease. Dysfunction of telomeres due to genetic abnormalities or age-related vulnerability of the DNA damage response can trigger senescence in AT2 cells, which contributes to dysfunctional repair [18]. Senescent AT2 cells, which are found in both IPF and CTD-ILD, exhibit impaired regenerative capacity [18,46] and develop a senescence-associated secretory phenotype (SASP), releasing proinflammatory and profibrotic mediators that activate macrophage and fibroblast [18]. In patients with systemic sclerosis-ILD, AT2 cells have significantly shorter telomeres than those from healthy controls [47], suggesting premature senescence as a contributor to these key disease features—epithelial dysfunction and fibrosis. Moreover, germline mutations in telomerase components such as *TERT* and *TERC*, which are well-established risk factors for familial pulmonary fibrosis, have also been identified in subsets of patients with CTD-ILD [48,49]. This finding suggests that telomere-mediated vulnerability contributes to a shared pathogenic substrate across ILD categories, including CTD-ILD.

In addition to cellular senescence, several genetic factors have been implicated in ILD susceptibility. The *MUC5B* promoter variant, a major risk allele for IPF, also confers increased susceptibility to RA-ILD [50,51], particularly in patients exhibiting a UIP pattern [52]. However, this association has not been observed in systemic sclerosis-ILD [53]. Variants in *TERT*, *RTEL1* and other telomere-maintenance genes have also been also implicated in susceptibility to ILD [48,54]. In RA-ILD, genetic determinants of RA itself—particularly *HLA-DRB1* shared epitope alleles and *PADI4* polymorphisms—may contribute to ILD susceptibility through heightened systemic inflammation and autoantibody production [10,55]. However, the evidence remains inconsistent, and the causal direction of these associations is not fully resolved [9,56,57].

Genetic factors play a crucial role not only in disease susceptibility but also in disease severity and prognostication. Variants in *SFTPA1/2*, *TOLLIP*, and short telomere length resulting from *TERT* mutation are associated with accelerated fibrosis and worse outcomes in ILD, although most data are derived from IPF studies [58,59,60]. Interestingly, while the *MUC5B* promoter variant increases genetic risk for ILD, it is paradoxically associated with slower disease progression and better survival, underscoring the complexity of genetic effects on disease behavior [61,62]. Despite these divergent effects on survival, these genetic determinants have drawn growing attention for their value in identifying ILDs with a “progressive phenotype” characterized by a propensity for subsequent fibrotic progression even without a definite radiologic UIP pattern. In line with these efforts, a ‘genomic classifier’ employing machine learning applied to next-generation RNA sequencing data has been developed [63]. This tool aims to detect molecular and histopathologic UIP signature at an early stage, thereby facilitating the timely initiation of antifibrotic therapy [63,64].

Collectively, these findings support a model in which genetic vulnerabilities impair epithelial repair capacity, lowering the threshold for injury and enabling environmental triggers to initiate a cycle of defective repair. In CTD-ILD, immune-mediated epithelial injury superimposed upon these inherent vulnerabilities may accelerate senescence and fibrinogenesis. These insights highlight the need for therapeutic strategies that protect the epithelium, restore its regenerative capacity, and utilize genomic profiling for early intervention.

### 2.2. Innate Immune Responses: Macrophage Polarization as a Bridge to Fibrosis

Alveolar macrophages serve as sentinel innate immune cells in the distal lung and play a pivotal role in the progression from injury to fibrosis [5]. Following epithelial damage, alveolar macrophages are activated by danger signals, such as extracellular ATP, unmethylated DNA, or hyaluronan fragments, via pattern recognition receptors [31]. Among these receptors, TLRs (particularly TLR2, TLR4, and endosomal TLR7/9) play a central role in initiating macrophage activation in ILD by sensing DAMPs released from injured epithelial cells [33,65,66]. Although direct evidence of TLR activation in CTD-ILD lung macrophages is limited, findings from other CTD contexts suggest that immune-complex uptake via Fcγ receptors can enhance endosomal TLR signaling [67,68]. This mechanism, while inferred, offers a biologically plausible explanation for how autoantibody-rich environments in CTD-ILD may amplify macrophage activation. Once activated, macrophages release a host of cytokines and chemokines that orchestrate downstream events.

#### 2.2.1. Macrophage Polarization: M1 vs. M2

A key concept in macrophage function is polarization, a process by which macrophages differentiate into specific functional phenotypes in response to various stimuli. Activated M1 macrophages are stimulated by interferon-γ (IFN-γ) and microbial signals [5]. They produce proinflammatory cytokines such as IL-1β, TNF-α, and IL-6, as well as chemokines that recruit neutrophils and lymphocytes [5]. While M1 macrophages contribute to inflammation in lung injury, alternatively activated M2 macrophages are implicated in fibrosis [41]. M2-polarized macrophages, which are induced by Th2 cytokines such as IL-4 and IL-13, secrete potent profibrotic factors, including TGF-β1 and platelet-derived growth factor (PDGF) [5,69]. STAT3 and STAT6 are also critical for M2 polarization [70,71]. M2 macrophages directly stimulate fibroblast activation and collagen deposition; they are a principal source of active TGF-β1, the master profibrotic cytokine that drives the differentiation of fibroblasts into myofibroblasts [5]. Through TGF-β and other mediators, M2-polarized alveolar macrophages can even induce epithelial cells to undergo epithelial–mesenchymal transition and become mesenchymal cells. Additionally, macrophages can produce growth factors and matrix metalloproteinases (MMPs) that remodel the extracellular matrix [72]. Thus, once macrophage polarization shifts toward the M2 phenotype, a profibrotic cascade is established.

In fibrotic ILDs, a clear skew toward the M2 phenotype has been observed in lung tissue and bronchoalveolar lavage fluid, even without active infection. M2 macrophages are predominant in the fibrotic lungs of both human patients and experimental models, and their accumulation is correlated with disease progression [69,73]. In CTD-ILD, the autoimmune milieu often contains abundant Th2 cytokines; for example, patients with systemic sclerosis-ILD have elevated serum levels of IL-4 and IL-13, which favor M2 polarization [74]. Studies have consistently reported the accumulation of profibrotic, M2-like CD206+ macrophages, which colocalize with areas of fibrosis, in lung biopsies from systemic sclerosis-ILD patients [75,76].

#### 2.2.2. Macrophages as a Dynamic Bridge to Fibrosis

The balance between proinflammatory (M1) and profibrotic (M2) macrophage activity is a critical determinant of disease trajectory [77]. Early in the disease course, M1 macrophages drive inflammation, which can cause symptoms and lung function impairment while also performing beneficial functions such as clearing debris. Over time, or with persistent injury, a cytokine-rich environment, such as that containing IL-4, IL-10, and IL-13, pushes macrophages toward the M2 phenotype, tipping the balance toward tissue remodeling and fibrosis.

Notably, circulating monocyte-derived macrophages supplement the pool of resident alveolar macrophages during disease. Blood monocytes, which are recruited to the injured lungs by chemokines such as CCL2, differentiate into macrophages or fibroblast-like cells [5]. In both IPF and systemic sclerosis-ILD, these monocyte-derived cells become fibroblast-like cells, which are a source of extracellular matrix components [78,79]. In anti-melanoma differentiation-associated gene 5 (anti-MDA5) dermatomyositis, circulating monocytes initially appear immunosuppressive but differentiate into monocyte-derived alveolar macrophages with profound inflammatory and fibrotic activity [80]. Single-cell RNA sequencing across various fibrotic ILDs revealed a population of monocyte-derived macrophages with distinct proinflammatory and profibrotic gene signatures, suggesting a common mechanism: alveolar injury leads to the infiltration of monocytes that mature into fibrosis-promoting macrophages, regardless of the initial trigger [81].

In summary, innate immune activation, particularly via alveolar macrophages, is a central driver of fibrogenesis in patients with CTD-ILD. Macrophages form a key bridge between initial injury and chronic fibrosis. They sense epithelial damage and are alternatively activated by the profibrotic type-2 environment, which in CTD-ILD is a consequence of the underlying autoimmune milieu. This same process is also a central feature of IPF. While macrophages drive fibrosis, the expanding fibrous tissue can, in turn, release damage signals that perpetuate macrophage activation, creating a self-reinforcing loop. This dynamic relationship underscores the rationale for therapeutic strategies that target macrophage function, such as the antifibrotic agent pirfenidone, which modulates macrophage polarization in an animal model [82,83], or Janus kinase (JAK) inhibitors, which interfere with the cytokine signaling pathways that drive this polarization [84].

### 2.3. Adaptive Immunity: The Autoimmune Engine of CTD-ILD

While innate immunity initiates the response, adaptive immune cells, such as T and B lymphocytes, play critical roles in sustaining chronic inflammation and autoimmunity in CTD-ILD. Adaptive immunity distinguishes CTD-ILD from IPF, and patients with CTD-ILD often present with lymphocytic infiltrates and tertiary lymphoid structures in their lungs, reflecting ongoing antigen-driven immune responses [85]. In this section, we discuss the actions of immune cells and their complicated interactions in the progression of CTD-ILD.

#### 2.3.1. T Cells: T Cell Dysregulation and Polarization

T cells play complex and multifaceted roles in the development and progression of fibrotic diseases, with different subsets promoting or inhibiting fibrogenesis [86,87]. The balance between these T-cell subsets and a skewing of T helper cell polarization are critical in determining the inflammatory and fibrotic trajectory [88].

##### T Helper 1 Cells: Antifibrotic Response

T helper 1 (Th1) cells, which are induced by IL-12 and are known for producing IFN-γ, primarily activate macrophages for microbial defense [88]. In the context of fibrosis, IFN-γ has distinct antifibrotic properties. It counteracts fibrosis by activating M1 macrophages and inhibiting collagen synthesis in fibroblasts through the upregulation of MMP expression [89]. IFN-γ also directly antagonizes the profibrotic effects of T helper 2 (Th2) cells by suppressing IL-13 production [90,91]. The importance of this antifibrotic role is underscored by studies showing that IFN-γ deficiency can increase IL-17 responses and lead to fibrosis [92]. Furthermore, natural killer T cells, which also produce IFN-γ, have been shown to protect mice from bleomycin-induced fibrosis by reducing Th2 cytokines and M2 macrophage polarization [93,94]. Therefore, while Th1 inflammation can cause tissue damage, a robust Th1 response appears to be essential for limiting. However, attempts to use IFN-γ therapeutically in IPF have not improved outcomes, suggesting that the timing and context of Th1 responses are critical [95].

##### T Helper 2 Cells: Profibrotic Drivers

In contrast to Th1 cells, Th2 cells are major drivers of fibrosis. Th2 cells, which are activated by IL-4, secrete IL-4, IL-5, and IL-13 and are primarily associated with humoral immunity and allergic inflammation [89]. A dominant Th2 response is strongly linked to both inflammation and fibrosis [96]. IL-13, a signature Th2 cytokine, is a potent inducer of fibrosis and promotes the alternative activation of M2 macrophages. It stimulates fibroblasts to differentiate and produce collagen and can even cause epithelial cells to undergo epithelial-mesenchymal transition. The profibrotic effect of IL-13 has been demonstrated in pulmonary fibrosis animal models, in which its overexpression is sufficient to cause fibrosis, whereas its knockout or blockade attenuates pulmonary fibrosis [97,98]. Similarly, IL-4 promotes M2 macrophage and fibroblast recruitment [99]. In CTD-ILD, particularly systemic sclerosis-ILD, a distinct Th2 skew is evident, with higher IL-4 levels in bronchoalveolar lavage fluid in systemic sclerosis-ILD than in that from nonfibrotic controls [100]. The profibrotic Th2 milieu not only directly causes fibrosis but also suppresses the protective Th1 responses that would otherwise keep fibrosis in check. While Romilkimab, which blocks IL-4 and IL-13, has been shown to effectively reduce dermal thickening in systemic sclerosis, it has not been shown to significantly improve lung function, underscoring the challenge of reversing fibrosis once established [101].

##### T Helper 17 (Th17) Cells: Sustaining Chronic Inflammation

Th17 cells, induced by IL-6 and TGF-β, produce IL-17 and IL-22 [102]. These cells are highly proinflammatory and are implicated in various autoimmune diseases [102]. In fibrotic ILD, IL-17A appears to enhance fibrosis by sustaining chronic inflammation and interacting with other pathways. It recruits neutrophils and stimulates epithelial and endothelial cells to produce more chemokines [102,103]. Furthermore, IL-17A can induce epithelial-mesenchymal transition by promoting TGF-β production and directly stimulating fibroblasts and fibrocytes [104]. Animal studies have shown that IL-17 knockout mice are protected from bleomycin-induced fibrosis and collagen deposition [105]. Both Th17 and Th2 cells are expanded in systemic sclerosis patients [106], indicating Th17-driven inflammation likely contributes to the chronic inflammatory environment and directly or indirectly promote fibrogenesis.

##### Regulatory T Cells and Their Complex Roles

Regulatory T cells (Tregs), which typically express FOXP3 and produce IL-10 and TGF-β, are generally responsible for suppressing autoimmunity [107,108]. Tregs play complex, context-dependent roles in CTD-ILD. While their primary function is restraining autoimmunity through IL-10 and latent TGF-β production, in the chronic IL-6 rich environment characteristic of CTD-ILD, Tregs become impaired—driven by FOXP3 instability, increased STAT3 phosphorylation, and reduced suppressive capacity—rather than undergoing conversion into proinflammatory effector phenotypes [109,110,111,112]. This functional deficit, not Treg mediated fibrosis, permits unopposed Th17 expansion and fibroblast activation [113,114].

##### Distinct T-Cell Dynamics in IPF vs. CTD-ILD

The distinct roles of T cells are further highlighted by the differences between IPF and CTD-ILD. In IPF, the absence of costimulatory molecules such as CD80/86 on fibroblasts and low expression of CD28 on T cells suggest a state of T-cell anergy or functional unresponsiveness [115,116]. In CTD-ILD, in contrast, the lung exists in a pro-inflammatory state with robust T-cell activation, likely because the systemic autoimmune milieu prevents such immune ‘escape’ phenomena. In vitro studies have shown that fibroblasts from IPF patients can secrete pro-apoptotic molecules that induce T cell apoptosis [116]. This apoptosis contributes to the observed lack of T cells in the fibrotic foci of the lung and supports the idea that the local environment created by fibroblasts is hostile to T-cell activation and survival, promoting functional anergy or deletion rather than a productive immune response.

Ultimately, in CTD-ILD, it is hypothesized that an imbalance between effector T cells (Th1/Th2/Th17) and Tregs permits autoimmunity to persist and drives the progression of fibrosis. This highlights a delicate immunological equilibrium in which a robust Th1 response might be protective, while a Th2/Th17 skew promotes fibrosis. Immunomodulatory therapies, therefore, should aim to restore this balance rather than merely suppress inflammation broadly.

#### 2.3.2. B Cells: B Cell Activation and Tertiary Lymphoid Structure Formation

Given their autoimmune nature, B cells and humoral immunity are central to the pathogenesis of CTD-ILD [117].

##### Autoantibodies and Their Pathogenic Role

Autoantibodies, a hallmark of CTD, are increasingly being implicated in lung pathology. In CTD-ILD, disease-specific autoantibodies (Table 1) may form immune complexes that deposit in pulmonary capillaries or alveoli, activating Fc receptors on macrophages and mast cells. For example, immune complexes containing rheumatoid factor can precipitate in the lung, inducing a localized immune response and leading to a neutrophil influx similar to that seen in the RA synovium [28].

In polymyositis and dermatomyositis, anti-tRNA synthetase antibodies (anti Jo1, PL-7, PL-12, and et al.) are the hallmark of antisynthetase syndrome. Autoantibodies against cytoplasmic aminoacyl-t RNA synthetases target synthetases expressed in muscle—and to a lesser extent in lung tissue—and are associated with an INF-γ related immune signature [118,119,120,121]. The pathogenesis of antisynthetase syndrome is thought to originate in the lungs, where environmental exposures and respiratory infections trigger an innate immune response [122,123]. This response leads to the cleavage of self-antigens, which in turn stimulates adaptive immunity and breaks down immune tolerance. This process culminates in the generation of specific T and B cells that produce characteristic antibodies, ultimately causing immune-mediated tissue damage and disease propagation in other organs [122].

**Table 1:** Clinical-immunologic features and immunopathogenesis of CTD-ILD subtypes.

CTD-ILD Subtype	Predominant ILD Pattern	Key Autoantibodies	Specific Immune/Cellular Features	Distinct Pathogenic Mechanisms
RA-ILD [28,124]	UIP	Anti-CCP antibody, Rheumatoid factor	Local protein citrullination, direct alveolar macrophage injury, TLS formation	Immune complex deposition, PAD enzymes, *MUC5B*-driven dysfunction, smoking-induced fibrotic microenvironment
SSc-ILD [30,125]	NSIP	ANAs, Anti-topoisomerase I	Activated fibroblast; Early B cell dysregulation, elevated IL-4/IL-13, TLS formation	Early vascular injury, endothelial dysfunction, alveolar epithelial injury, profibrotic mediators from epithelial cells, TGF- β dominant fibrotic loop
Myositis-ILD [126,127]	NSIP	Anti-tRNA synthetase, anti-MDA5, anti-Ro52 antibodies	Hyperinflammatory alveolitis (anti-MDA5), type I IFN induction	tRNA-synthetase antigen recognition in lung, IFN-driven macrophage injury,
Sjögren’s-ILD [16]	NSIP	Anti-SSA/Ro, anti-SSB/La antibodies	TLS formation, B cell activation	B cell-driven autoimmunity and lymphoid neogenesis
Mixed Connective Tissue Disease-ILD [128,129]	NSIP	Anti U1 RNP	Type 1 IFN signature, nucleoprotein immune complex, plasmacytoid dendritic cell activation	U1 RNP immune complex, IFN-I response, endothelial dysfunction/ vasculopathy, fibroblast activation.

ANA: Antinuclear antibodies; Anti-CCP: Anti-cyclic citrullinated peptide; Anti-MDA5: Anti- melanoma differentiation-associated gene 5; ILD: Interstitial lung disease; IFN: Interferon; NSIP: Nonspecific interstitial pneumonia; PAD: peptidyl arginine deiminase; RA: Rheumatoid arthritis; SSc: Systemic sclerosis; TLS: Tertiary lymphoid structure; UIP: Usual interstitial pneumonia.

A particularly aggressive example is anti-MDA5 antibodies, associated with rapidly progressive ILD in amyopathic dermatomyositis. These antibodies likely form immune complexes that stimulate macrophages via Fcγ receptors and strongly induce type I IFN from plasmacytoid dendritic cells [126]. This phenomenon fuels hyperinflammatory alveolitis that can quickly lead to acute respiratory distress syndrome. While type I IFN is a key cytokine in anti-MDA5 dermatomyositis and lupus pneumonitis, in systemic sclerosis-ILD, evidence points to significant inflammation and endothelial injury contributing to vasculopathy, although vasculopathy can manifest in all of these diseases [130]. Therapeutic strategies targeting this pathway are emerging. Direct IFN-I blockade with anifrolumab is biologically compelling in anti-MDA5 dermatomyositis-ILD, but clinical evidence is limited to case reports [131]. In contrast, JAK inhibitors, which attenuate IFN-I signaling at the IFN-α/β receptor-JAK1/TYK2 node, currently have stronger real-world and cohort-level support in anti-MDA5 ILD [132].

Anti-Ro52 (TRIM21) is another antibody associated with ILD, particularly in inflammatory myositis and anti-MDA5 syndrome. Its presence is associated with more severe ILD phenotypes and worse outcomes [16,133]. Anti-Ro 52 positivity has been identified as a risk factor for developing ILD in patients with Sjogren syndrome and inflammatory myositis [134]. Notably, the coexistence of anti-ro 52 antibody and myositis specific autoantibodies often indicated a high-risk subset with poor prognosis, suggesting a possible additive or synergistic effect [135]. Anti Ro 52 antibodies contribute to ILD via direct pathogenic effects, such as immune complex formation and Fcγ receptor engagement on macrophages, as well as through the impairment type I IFN regulation. Given that Ro52 normally suppress type I IFN through ubiquitin-mediated degradation of IRF-7, the functional dysregulation of this protein permits unopposed type I IFN signaling [133,136,137].

The anti-topoisomerase I antibody (anti-SCL70) is the primary serologic maker associated with the diffuse cutaneous type of systemic sclerosis and is strongly predictive of ILD [138], which tends to follow a more aggressive course in these patients. These antibodies recognize nuclear topoisomerase and reflect an underlying immune response in which topoisomerase-1-reactive T cells attack multiple tissues including the lung [139,140]. Their presence marks a highly activated immune state characterized by the expansion of autoreactive B cell responses, and is strongly associated with ILD in systemic sclerosis [134,139,141]. Immune complexes containing topoisomerase I have been proposed to enhance antigen presentation, engage the TLR-mediated innate pathway, and promote fibroblast activation in TGF-β-dominant milieu [139,142]. These processes could contribute to ongoing inflammation and fibrosis. Anti U1 RNP is a serologic marker of mixed connective tissue disease, and it promotes nucleoprotein-containing immune complex formation and activate the IFN-1 pathway via TLR engagement [128].

In CTD-ILD, autoantibodies are not just diagnostic biomarkers, but also participate actively in immunopathogenic pathogenesis in multiple ways. They link systemic autoimmunity to lung injury through immune complex deposition, complement activation, and stimulate fibroblast via cytokine crosstalk. The presence of certain autoantibodies in early stage CTD-ILD can precede clinical symptoms, prompting the close monitoring of ILD progression [143,144].

##### B Cells as Antigen-Presenting and Cytokine-Producing Cells

In addition to producing autoantibodies, B cells act as antigen-presenting cells and cytokine producers that modulate T-cell responses and the fibrosis cascade. In systemic sclerosis, B-cell dysregulation is an early event, and B cells infiltrate target organs such as the skin and lungs.

A key feature of CTD-ILD is the formation of tertiary lymphoid structures in the lungs. These structures resemble germinal centers, with follicular dendritic cells, distinct B-cell follicles, and T-cell zones [145,146]. Within these ectopic lymphoid tissues, B cells undergo activation and somatic hypermutation, which are supported by the local production of lymphoid-organizing chemokines such as CXCL13 and survival factors such as B-cell activating factor (BAFF) [145,146]. The presence of such lymphoid aggregates in RA and Sjögren’s syndrome has been correlated with more severe tissue damage, indicating that in situ autoimmunity can aggravate fibrosis [147]. While lymphoid aggregates can be found in IPF, they are often less organized and lack the robust follicular dendritic cell networks seen in CTD-ILD [147]. These findings suggest that while immune cells may be present in IPF, the local environment is not conducive to a sustained, organized adaptive immune response. Tertiary lymphoid structures suggest that the adaptive immune response in CTD-ILD is driven by a vicious cycle of local inflammation and a highly organized immune response occurring directly within the lung tissue, a cycle that is sustained by a systemically primed immune response. Studies of bronchoalveolar lavage fluid from CTD-ILD patients have shown an enrichment of IgG-positive memory B cells and FCRL5^+^ (Fc receptor-like 5 positive) B cells, suggesting chronic antigen exposure [148].

The BAFF/BLyS pathway is particularly noteworthy, as elevated BAFF levels are found in systemic sclerosis-ILD patients and are correlated with more severe lung fibrosis and reduced survival [149]. Serum BAFF levels have been shown to be higher in CTD patients than in healthy subjects and to differ between ILD patients and non-ILD patients [150]. BAFF overproduction leads to hyperactive B cells that can disrupt immune tolerance. In a systemic sclerosis animal model, BAFF inhibition has been shown to attenuate fibrosis by reducing autoantibody levels and, importantly, by modulating cytokine profiles—specifically suppressing fibrogenic cytokines and increasing antifibrotic IFN-γ levels [151,152]. These findings indicate that B cells contribute to fibrosis via cytokine secretion as much as antibody production does.

In summary, adaptive immunity provides a persistent, antigen-driven inflammatory environment that perpetuates lung damage and fibrosis in CTD-ILD. While Th2 and Th17 CD4^+^ T cells skew the immune response toward chronic inflammation and fibrogenesis, B cells contribute by producing autoantibodies and fibrogenic cytokines and by organizing into tertiary lymphoid structures that localize immune attacks within the lungs. Because of the multifaceted role of B cells, B-cell-targeting therapies such as rituximab or BAFF inhibitors can have pleiotropic effects, impacting both humoral and cellular immunity and potentially leading to broader antifibrotic benefits [153]. The demonstrated efficacy of rituximab in clinical trials for treating CTD-ILD, including systemic sclerosis [154,155], lends support to this idea, suggesting that rituximab can disrupt the autoimmune cascade at multiple levels and attenuate the inflammatory–fibrotic feedback loop. In the future, even more potent B-cell-depleting agents, such as obinutuzumab [156], could be evaluated in the CTD-ILD field. In contrast to that in CTD-ILD, the adaptive immune response in IPF is often suppressed and lacks the vigorous autoimmune activation observed in CTD-ILD. The immunopathogenesis of CTD-ILD involves numerous interconnected signaling pathways that link inflammation and fibrosis, as summarized in the table below (Table 2).

### 2.4. Fibroblast Activation and the Progression from Inflammation to Fibrosis

The final common pathway in ILD pathogenesis is the activation of fibroblasts and their differentiation into myofibroblasts. It leads to the excessive deposition of extracellular matrix in the lung interstitium [14]. Persistent immune activation, as described previously, creates a microenvironment rich in fibrogenic signals, including TGF-β, PDGF, IL-6, and IL-13, which drive fibroblast activation [157,164,165]. In healthy response to acute injury, fibroblasts transiently proliferate and lay down the matrix for tissue repair, subsequently undergoing apoptosis or returning to quiescence. However, in CTD-ILD, this process becomes dysregulated: fibroblasts escape normal feedback control and continue to proliferate and secrete collagen, even as inflammation persists [166]. Systemic sclerosis is a representative disease in which fibroblasts are aberrantly active in various organs, including the lungs.

#### 2.4.1. TGF-β: The Master Regulator of Fibrosis

TGF-β, particularly TGF-β1, is a potent cytokine involved in fibrogenesis [167]. It plays an integral role in the development of fibrosis, a process involving the excessive formation of scar tissue. Almost all cell types in the lungs, including epithelial cells, macrophages, T cells, and fibroblasts, can produce latent TGF-β. Latent TGF-β is then activated by integrins or proteases, allowing it to initiate a cascade of events [114]. Once activated, TGF-β binds to its receptor on fibroblasts and signals through the SMAD pathway, specifically SMAD2/3 [168,169]. This signaling alters gene transcription, leading to the upregulation of the expression of genes that produce key components of the extracellular matrix, such as collagen (*COL1A1* and *COL3A1*) and fibronectin [170,171]. Furthermore, it induces differentiation into myofibroblasts, a process marked by the elevated expression of α-smooth muscle actin [172].

Myofibroblasts are pathological fibroblasts with smooth muscle-like features, which enable them to generate tension and contract tissue. This contractility contributes to the architectural distortion seen in fibrotic lungs. Myofibroblasts are also highly synthetic and produce large amounts of extracellular matrix. In biopsies from fibrotic ILD, clusters of myofibroblasts, known as “fibroblast foci,” are found in areas of active fibrosis and are considered the drivers of scar formation [173].

The role of TGF-β extends beyond stimulating fibroblasts. TGF-β also influences other cell types and pathways that contribute to fibrosis. For example, in vitro studies have shown that TGF-β induces IL-6 expression in human bronchial epithelial cells through the SMAD2/3 pathway [174] and TGF-β. In CTD-ILD, particularly in systemic sclerosis-ILD and sometimes in myositis-related ILD, the TGF-β/SMAD axis is aberrantly activated [175]. In patients with systemic sclerosis, TGF-β has even been shown to increase IL-13 synthesis in T lymphocytes, further highlighting its broad impact on immune regulation and fibrosis processes [176].

While TGF-β is a primary therapeutic target for fibrosis, directly inhibiting it has proven challenging. Clinical studies have not yet demonstrated clinical efficacy, largely because of the widespread role of TGF-β in immune regulation and tissue homeostasis [157,177]. Despite these challenges, the central role of TGF-β in driving fibrosis remains a key area of research.

#### 2.4.2. PDGF, FGF and VEGF Signaling

PDGF is released by activated macrophages, damaged alveolar epithelium, and platelets and acts as a potent chemoattractant and mitogen in fibrotic lung diseases [178]. The PDGF A and PDGF B isoforms and their receptors PGDFR-α/β are upregulated in lung fibrosis, and drive fibroblast proliferation, migration, and collagen synthesis [178]. Excessive connective tissue deposition in the interstitial space by myofibroblasts eventually results in a distorted alveolar structure with compromised gas exchange. In systemic sclerosis, the level of PDGF is elevated in BAL fluid [179], and stimulatory autoantibodies targeting PDGFR can induce reactive oxygen species and collagen production, linking autoimmunity to PDGF-driven fibroblast activation [180].

Fibroblast growth factor (FGF) promotes fibroblast growth, survival, and extracellular matrix production and it can be produced by alveolar macrophages, fibroblasts, T-lymphocytes, and endothelial cells [181,182]. Elevated FGF-2 levels have been detected in serum and skin in patients with systemic sclerosis [183,184]. PDGF and FGF activate receptor tyrosine kinases such as PDGFR-α/β, FGFR 1-3 that signal via PI3K/AKT and RAS-RAF-MAPK, converging on transcriptional programs that promote matrix deposition and fibroblast resistance to apoptosis [185]. The FGF/FGF receptor (FGFR) signaling cascade is regulated by TGF-β, illustrating the extensive interconnection among various growth factors [186,187].

Vascular endothelial growth factor (VEGF) contributes to vascular remodeling as well as fibrinogenesis in CTD-ILD, especially in systemic sclerosis [188]. Endothelial injury triggers VEGF release, lead to aberrant angiogenesis, endothelial to mesenchymal transition, and fibroblast accumulation. Increased VEGF expression from systemic sclerosis highlights the importance of endothelial-fibroblast crosstalk in autoimmune fibrogenesis [189].

The clinical efficacy of nintedanib, a tyrosine kinase inhibitor targeting PDGFR, FGFR, and VEGRF [190], across IPF [191], systemic sclerosis-ILD [192], and progressive fibrosing CTD-ILD [193] underscores the centrality of these interconnected growth-factor pathways in autoimmune-driven lung fibrosis. Collectively, PDGF, FGF, and VEGF form an integrated fibrotic and angiogenetic network that sustain fibroblast activation, vascular remodeling, and progressive scarring in CTD-ILD.

#### 2.4.3. IL-6

IL-6 is a pleiotropic cytokine with both immunologic and profibrotic effects. In systemic sclerosis-ILD, IL-6 directly activates fibroblasts via JAK/STAT3 signaling, promoting type I collagen synthesis, myofibroblast differentiation, resistance to apoptosis, and reduced MMP-mediated collagen degradation [158,194]. IL-6 trans-signaling through soluble IL-6R further amplified these responses by sustaining STAT3 activation in fibroblasts and other structural cells [158]. Blockade of IL-6 trans-signaling attenuated bleomycin pulmonary fibrosis in vivo [158,195]. Consistently, animal models showed that overexpression of IL-6 augmented fibrotic response to bleomycin [165], whereas genetic or pharmacologic interruption of IL-6/STAT3 signaling reduced fibrosis, particularly in the chronic phase of injury [196].

Clinically, high serum IL-6 levels in early systemic sclerosis-ILD predict a decline in lung function and increased mortality, suggesting that IL-6 is a biomarker of the immune-fibrotic phenotype [197]. Similar associations have been reported in anti-MDA 5 dermatomyositis-ILD, where elevated IL-6 independently predicts rapidly progressive ILD and poor prognosis [198]. These data support a model in which IL-6 is highly active in early inflammation stages but continues to drive fibroblast activation and tissue remodeling during chronic disease, especially in autoimmune-related ILD where inflammation and fibrosis coexist for prolonged period.

This biology makes IL-6 an attractive therapeutic target. Tocilizumab, which blocks both membrane-bound and soluble IL-6 R, has demonstrated the preservation of lung function in ILD in patients with early systemic sclerosis in randomized trials and post hoc analysis [199,200]. In RA-ILD, retrospective cohort studies of IL-6 receptor inhibitors such as tocilizumab and sarilumab have suggested that IL-6/STAT3 blockade can control synovial inflammation without worsening ILD [201,202], although robust controlled data are limited. Taken together, the data suggest that IL-6 not only fuels Th17 and B cell activation but also acts directly on fibroblast and even alveolar epithelial cells, effectively bridging immune activation and fibrinogenesis.

#### 2.4.4. IL-13 and IL-17

Beyond polarizing macrophages, IL-13 acts on fibroblasts through IL-13 receptors, thereby activating STAT6 [203]. Th2-biased conditions with high IL-13 levels often result in subepithelial fibrosis, underscoring the fibrogenic capacity of IL-13 [89]. IL-17A stimulates fibroblasts to produce IL-6, IL-8, and GM-CSF, perpetuating inflammation. IL-17–driven neutrophil recruitment and neutrophil extracellular traps may contribute to lung damage in patients with RA [28,204] and systemic sclerosis [205,206]. Some data suggest that IL-17 can also cooperate with TGF-β to promote epithelial–mesenchymal transition via STAT3 signaling [104].

#### 2.4.5. JAK/STAT Signaling: Integrating Inflammatory and Fibrotic Signals

Many cytokines central to CTD-ILD pathogenesis—including IL-6, IL-13, IFN-γ, and GM-CSF—signal through JAK family kinases and activate downstream STAT transcription factors, particularly STAT3 and STAT6 [158,162]. In the context of CTD-ILD, the JAK/STAT pathway integrates both proinflammatory and profibrotic cues: IL-6/STAT3 signaling promotes Th17 differentiation, fibroblast survival, and collagen production, whereas IL-13/STAT6 contributes to M2 macrophage polarization and matrix remodeling [207]. Together, these cytokine-driven programs create a sustained immune-fibrotic circuit that is characteristic of CTD-ILD.

JAK/STAT inhibition appears to control inflammation without worsening lung function, making it a promising dual-target strategy for selected CTD-ILD subsets, driven by clinical observations in anti-MDA5 dermatomyositis and encouraging results in refractory myositis-associated ILD [132]. In vitro analyses have revealed that treatment with baricitinib, a JAK1/2 inhibitor, attenuates epithelial–mesenchymal transition in alveolar epithelial cells incubated with IL-6 [208]. As the understanding of cytokine cross-talk deepens, the JAK/STAT pathway has been shown to represent a mechanistically compelling and therapeutically actionable axis in immune-driven fibrotic lung disease. Although JAK/STAT activation—particularly STAT3 phosphorylation in epithelial and fibroblast compartments—is demonstrable in IPF [209], it is not the dominant upstream driver, a role played instead by the TGF-β/SMAD, epithelial senescence, and epithelial stress pathways. In contrast, the pathogenesis of CTD-ILD is tightly linked to cytokine-rich inflammatory circuits involving IL-6, IL-13, GM-CSF, and type I IFN, making JAK/STAT a more central and therapeutically tractable pathway. Recently published ERS/EULAR recommendations acknowledge JAK inhibitors as a potential option in RA-ILD and myositis-ILD treatment, reflecting growing but still limited evidence and emphasizing individualized risk–benefit assessment [210].

#### 2.4.6. PDE4: Modulating Immune-Fibrotic Crosstalk

Phosphodiesterase 4 (PDE4) is an intracellular enzyme that is highly expressed in immune cells and fibroblasts and is responsible for degrading cyclic adenosine monophosphate (cAMP), a second messenger with broad anti-inflammatory and antifibrotic effects. In the lung, increased PDE4 activity contributes to a proinflammatory and profibrotic environment by lowering cAMP levels, thereby enhancing inflammatory cytokine production, fibroblast activation, and TGF-β signaling sensitivity [211,212]. Through its regulation of intracellular signaling, PDE4 acts as a key modulator of immune-fibrotic crosstalk, influencing both macrophage polarization and fibroblast responsiveness to fibrogenic stimuli. In a systemic sclerosis animal model, the PDE inhibitor nerandomilast has been shown to alleviate skin and lung fibrosis through the inhibition of PDE and consequent dampening of the TGF-β pathway [213].

In the recent FIBRONEER-ILD phase 3 trial, the PDE4B inhibitor nerandomilast met its primary endpoint by slowing forced vital capacity decline at 52 weeks in patients with progressive pulmonary fibrosis [211]. Intriguingly, exploratory analyses revealed that the 18 mg dose showed greater benefit in the CTD-ILD subgroup, suggesting that the anti-inflammatory effects of nerandomilast might be more significant than its antifibrotic effects, thereby reemphasizing the key role of inflammation in the pathogenesis of CTD-ILD [211].

#### 2.4.7. TNF-α and IL-1β

TNF-α and IL-1β are acute-phase proinflammatory cytokines mainly produced by macrophages. In chronic conditions, these cytokines can also induce fibroblasts to express adhesion molecules and chemokines, thereby recruiting more fibroblasts and inflammatory cells [214]. While clinical trials targeting TNF-α in IPF have failed [215], suggesting a lack of benefit in that context, TNF-α inhibitors are widely used for CTDs such as RA. Therefore, it is crucial to understand how these targeted therapies affect not only the primary affected organs, such as the joints, but also the lungs in patients with ILD to guide a tailored treatment approach.

#### 2.4.8. Chemokines and Other Profibrotic Molecules

Fibroblasts themselves secrete chemokines, such as CCL2, CCL7, and CXCL12, which attract monocytes and more fibrocytes, amplifying the fibroinflammatory loop [216]. CCL18, produced by alveolar macrophages, particularly in ILD, is both a biomarker and a player that can induce collagen production by fibroblasts and is elevated in the serum of patients with progressive fibrosis [217,218]. Notably, the same profibrotic molecules may function somewhat differently in IPF and CTD-ILD. For example, in IPF, the pathogenic footprint of heat shock protein 90 aligns with an epithelial-senescence and fibroblast-autonomous program—stabilizing profibrotic signaling (e.g., TGF-β/SMAD, MAPK) within self-sustaining fibroblastic niches [219]. In CTD-ILD, the same chaperoning functions occur but are embedded within an immune-rich milieu; thus, heat shock protein 90 acts as a stress transducer that couples epithelial injury to both innate cascades (NF-κB-dependent) and fibroblast activation [220].

In summary, these mediators form a complex network that drives fibrosis. This reflects how the mediators involved in fibrosis are also involved in multiple steps of inflammation and immunity, highlighting the intertwined processes of inflammation, immunity, and fibrosis. Importantly, fibroblasts in the fibrotic lung not only form scars but also interact with immune cells, indicating that therapies dampening fibroblast activity can indirectly modulate inflammation. The failure of single-cytokine targeting, such as anti-TNF targeting, in IPF suggests that effective antifibrotic strategies may be needed to affect multiple pathways of the network. Medications such as nintedanib, a multikinase inhibitor, or JAK inhibitors, which broadly affect many cytokine signals, represent this kind of multitarget approach. The interplay of immune and fibrotic pathways in CTD-ILD underscores why combination therapy addressing both arms is biologically rational.

## 3. Spectrum of CTD-ILD and IPF

While CTD-ILD and IPF share a common final pathway of fibrosis, their underlying pathogenic drivers are distinct. IPF is primarily an epithelial-centric disease of dysfunctional repair in a genetically susceptible, aging host [221]. In contrast, CTD-ILD is fundamentally an immune-driven condition in which systemic autoimmunity initiates and sustains epithelial injury. This process involves the synergistic interaction of innate and adaptive immunity, ultimately leading to a self-perpetuating fibrotic loop. This conceptual distinction was reinforced by the PANTHER trial, which demonstrated that immunosuppressive therapy, including corticosteroids, was associated with increased risks of death and hospitalization in IPF patients, highlighting a fundamental difference in disease pathogenesis [222]. These findings led to the hypothesis that unlike the inflammation-driven, self-perpetuating fibrotic pathway in CTD-ILD, IPF involves a distinct, inflammation-independent fibrogenic pathway.

Distinct cytokine signatures among CTDs influence the development, severity, and inflammatory versus fibrotic nature of ILD (Table 1). In immune-dominant phenotypes—such as those associated with idiopathic inflammatory myopathies, Sjögren’s syndrome, and mixed connective tissue disease—adaptive immunity drives lung injury through B-cell activation, autoantibody production, type I IFN signaling, and tertiary lymphoid structures. These subtypes typically manifest the NSIP pattern, progress more slowly than IPF does, and respond to immunomodulatory therapy. At the fibrosis-dominant end of the spectrum, systemic sclerosis itself and systemic sclerosis-ILD exhibit constitutive fibroblast activation and TGF-β-driven extracellular matrix production early in the disease course. Although the most common histological pattern is NSIP rather than UIP, this shared profibrotic program with IPF provides a rationale for early antifibrotic therapy, which has demonstrated clinical benefit [192,223]. RA-UIP, which has fibroblastic foci and a MUC5B promoter variant, can drive a more fibrotic phenotype [9]. This is especially true in patients with a history of smoking and high titers of autoantibodies [224]. Despite these similarities to IPF, RA-UIP retains a more significant lymphocytic infiltrate in lung tissue [147], reflecting its autoimmune context. Interestingly, in an RA-ILD animal model, nintedanib has been shown to reduce the expression of PAD and citrullinated peptides in the bronchoalveolar epithelium, suggesting that it ameliorates mucosal injury [225]. Although both CTD-ILD and IPF converge on fibroblast activation and progressive fibrosis, their upstream cytokine milieus diverge: CTD-ILD is characterized by adaptive immune skewing with heightened type I IFN signatures, BAFF-mediated B-cell survival, the JAK/STAT pathway, and IL-6–driven inflammation, whereas IPF is characterized by type-2 inflammation and downstream IL-13/IL-4–M2 macrophage loops.

The term “inflammation–immunity–fibrosis continuum” indicates that there is no sharp boundary at which inflammation ends and fibrosis begins; rather, immunological and fibrotic processes feedback on each other [8]. However, this continuum operates differently in IPF and CTD-ILD. IPF is primarily an epithelial-centric disease driven by a dysfunctional repair response, and the fibrotic process is thought to become autonomous and even actively suppress adaptive immune responses. In contrast, CTD-ILD is fundamentally an immune-driven pathology. The initial insult is not merely an epithelial defect but also an autoimmune attack mediated by T cells or autoantibodies that directly target the alveolar epithelium. This systemic autoimmune milieu is the true engine of the disease, and this persistent, antigen-driven inflammation primes the lung environment for fibrosis. In CTD-ILD, fibroblasts are reactive players and are continuously stimulated by the chronic inflammatory state. This difference provides the mechanistic basis for why CTD-ILD often responds to immunomodulatory therapies, whereas broad immunosuppression is not very beneficial in IPF. In CTD-ILD, the fibrotic phase can dominate if inflammation is not controlled early. Notably, fibrosis can become self-sustaining even if the initial autoimmune trigger is removed or immunosuppression is applied. This observation highlights the shift from an immune-driven inflammatory phase to a predominantly fibrotic pathology that is less responsive to immunotherapy. This explains the current clinical paradigm of often using both immunosuppressants and antifibrotics to treat CTD-ILD, as early intervention is critical, but for advanced disease, antifibrotic drugs are indispensable [226,227].

The latest clinical practice guidelines from the American Thoracic Society (ATS) for Systemic Sclerosis-ILD [223] and the American College of Rheumatology/American College of Chest Physicians for systemic autoimmune rheumatic disease-ILD [228] provide a formal, evidence-based framework for treatment, reinforcing the use of immunomodulatory agents such as mycophenolate and rituximab and underscoring the importance of antifibrotic therapy for progressive fibrotic phenotypes. This paradigm shift toward antifibrotic treatment for CTD-ILD and other non-IPF ILDs was solidified by the results of the INBUILD trial, a pivotal study showing that the antifibrotic drug nintedanib effectively attenuated the rate of forced vital capacity decline in a diverse group of patients with progressive fibrosing ILDs, a cohort that included a significant subset of patients with CTD-ILD [193].

The commonalities between CTD-ILD and IPF in later stages suggest that many fibrotic pathways converge regardless of the initial cause. Indeed, progressive fibrosing ILDs of various etiologies respond to antifibrotic drugs, reinforcing the idea of shared fibrotic mechanisms. In light of this understanding and in light of the findings of the INBUILD trial, the 2022 ATS/ERS/JRS/ALAT clinical practice guidelines introduce the concept of progressive pulmonary fibrosis to capture the common clinical trajectory observed across patients with fibrotic ILDs [229]. This framework shifts the focus from disease etiology alone to a phenotypic lens of progression, recognizing that non-IPF ILDs can follow a course closely resembling IPF. These findings underscore the importance of antifibrotic therapy, similar to IPF management, in addition to immunosuppressive agents.

## 4. Conclusions

CTD-ILD lies at the intersection of autoimmunity and fibrosis and involves complicated crosstalk among injured alveolar epithelial cells, innate immune cells, and adaptive immunity.

CTD-ILD research is rapidly evolving and driven by technological advances in single-cell genomics and systems immunology. Results from such studies are revealing the remarkable cellular and molecular complexity of autoimmune lung disease and identifying new therapeutic targets. Several key questions remain to be answered. The mechanisms that determine why certain CTD patients develop ILD while others do not remain poorly understood. The role of environmental triggers, such as viral infections or particulate exposure, in initiating or exacerbating autoimmune lung disease requires further investigation. The optimal timing and sequencing of immunosuppressive and antifibrotic therapies need to be better defined through prospective clinical trials, complex biomarker analyses, and quantitative imaging studies.

The ultimate goal is to transform CTD-ILD from a condition with high morbidity and mortality to a manageable chronic disease. Meeting that goal will require continued collaboration among rheumatologists, pulmonologists, and immunologists to translate mechanistic insights into effective treatments. The distinct immunopathogenic features of CTD-ILD provide both challenges and opportunities—challenges in the multidirectional interplay of the pathogenesis but also opportunities in the multiple potential therapeutic targets that this complexity reveals.

As our understanding of the inflammation-immunity-fibrosis continuum continues to evolve, we move closer to truly personalized therapy for CTD-ILD patients. Ongoing research will likely refine these approaches, potentially introducing novel agents that specifically target pathways [164,230]. By matching treatment strategies to the dominant pathogenic mechanisms in individual patients, we can optimize outcomes and minimize treatment-related toxicity. It also underscores the enduring importance of a collaborative, patient-centered, and multidisciplinary care team. The future of CTD-ILD management lies in this precision medicine approach, guided by a deep understanding of the immunopathogenic mechanisms that drive this complex group of diseases.

## Figures and Tables

**Table 2:** Key molecular mediators, roles in pathogenesis, targeted therapies, and associated CTDs.

Mediator	Main Source	Role in Pathogenesis	Targeted Therapy	Associated CTDs
TGF-β [157]	M2 macrophages and epithelial cells	Master profibrotic cytokine; induces fibroblast activation and ECM deposition via SMAD pathway and EMT	No approved direct therapy; hard to block safely	SSc-ILD and myositis-ILD
IL-6 [158]	Macrophages, T cells, and B cells	Promotes inflammation, Th17 differentiation, and fibroblast proliferation via STAT3	Tocilizumab (anti-IL-6R)	SSc-ILD and RA-ILD
IL-13 [159]	Th2 cells	Drives M2 macrophage polarization, fibroblast activation, and EMT via STAT6	Experimental (e.g., anti-IL-13 antibodies and IL-4Rα blockers)	SSc-ILD
IL-17 [104]	Th17 cells and γδ T cells	Sustains chronic inflammation and synergizes with TGF-β to induce fibrosis via STAT3	Under investigation (an anti-IL-17A antibody is not approved for ILD)	SSc and myositis-ILD
IFN-γ [88]	Th1 cells and NKT cells	Counterbalances Th2 and Th17 responses; generally, antifibrotic	Not currently used; past trials unsuccessful in IPF	RA and general antifibrotic role
BAFF [160]	B cells and dendritic cells	Promotes B cell survival, autoantibody production, and cytokine release (IL-6, IL-10)	Belimumab (anti-BAFF antibody; used in SLE; and explored in SSc)	SSc-ILD, RA-ILD, and Sjogren’s syndrome
CXCL13 [161]	Follicular dendritic cells and macrophages	Drives tertiary lymphoid structure formation and recruits B and Tfh cells	None yet; potential biomarker and research target	RA-ILD and Sjogren’s syndrome
JAK/STAT [162]	Downstream of IL-6, IL-13, and IFN-γ	Integrates multiple cytokine signals and drives pro-fibrotic immune skewing	JAK inhibitors (tofacitinib, baricitinib, and filgotinib)	RA-ILD and myositis-ILD
Citrullinated Proteins [28]	Smoking-induced lung cells (RA) and PAD enzymes	Acts as DAMPs, promoting epithelial injury and triggering anti-CCP antibody production	No direct therapy; linked to smoking cessation and autoantibody screening	RA-ILD
PDGF (Platelet-Derived Growth Factor) [163]	Activated macrophages, platelets, injured epithelial cells, and fibroblasts	Stimulates fibroblast proliferation, chemotaxis, survival, and ECM production and promotes myofibroblast differentiation and tissue remodeling	Nintedanib (tyrosine kinase inhibitor that blocks PDGFR, FGFR, and VEGFR)	CTD-ILD with progressive fibrosis SSc-ILD, and RA-ILD

BAFF: B cell activating factor; DMAP: Damage-associated molecular patterns; ECM: Extracellular matrix; EMT: Epithelial-mesenchymal transition; FGFR: Fibroblast growth factor receptor; ILD: Interstitial lung disease; NKT: Natural killer T cells; PAD: Peptidyl arginine deiminase; PDGFR: Platelet-derived growth factor receptor; RA: Rheumatoid arthritis; SSc-ILD: Systemic sclerosis-associated ILD; STAT: Signal transducer and activator of transcription; Th2 cells: T helper 2 cells; Tfh cells: T follicular helper cells; VEGFR: Vascular endothelial growth factor receptor.

## Data Availability

No new data were created or analyzed in this study. Data sharing is not applicable to this article.
